# The Relationship Between Impulsivity Traits and In Vivo Cerebral Serotonin Transporter and Serotonin 2A Receptor Binding in Healthy Individuals: A Double-Tracer PET Study with C-11 DASB and C-11 MDL100907

**DOI:** 10.3390/ijms26010252

**Published:** 2024-12-30

**Authors:** Jeong-Hee Kim, Hang-Keun Kim, Young-Don Son, Jong-Hoon Kim

**Affiliations:** 1 Biomedical Engineering Research Center, Gachon University, Incheon 21936, Republic of Korea; 2 Department of Biomedical Engineering, College of IT Convergence, Gachon University, Seongnam-si 13120, Republic of Korea; 3 Neuroscience Research Institute, Gachon University, Incheon 21565, Republic of Korea; 4 Department of Psychiatry, Gachon University College of Medicine, Gil Medical Center, Gachon University, Incheon 21565, Republic of Korea

**Keywords:** impulsivity, positron emission tomography, serotonin transporter, serotonin 2A receptor, caudate

## Abstract

To elucidate the potential roles of presynaptic and postsynaptic serotonergic activity in impulsivity traits, we investigated the relationship between self-reported impulsiveness and serotonin transporter (5-HTT) and 5-HT2A receptors in healthy individuals. In this study, 26 participants completed 3-Tesla magnetic resonance imaging and positron emission tomography with [^11^C]DASB and [^11^C]MDL100907. To quantify 5-HTT and 5-HT2A receptor availability, the binding potential (BP_ND_) of [^11^C]DASB and [^11^C]MDL100907 was derived using the simplified reference tissue model with cerebellar gray matter as the reference region. The participants’ impulsivity levels were assessed using the Barratt Impulsiveness Scale-11 (BIS-11). The region of interest (ROI)-based partial correlation analysis with age, sex, and temperament traits as covariates revealed a significant positive correlation between non-planning impulsiveness and [^11^C]MDL100907 BP_ND_ in the caudate (CAU) at Bonferroni-corrected *p* < 0.0045. Non-planning impulsiveness was also positively correlated with [^11^C]MDL100907 BP_ND_ in the prefrontal cortex (PFC), ventromedial PFC, orbitofrontal cortex (OFC), insula (INS), amygdala (AMYG), putamen, ventral striatum, and thalamus, and the total score of BIS-11 was positively correlated with [^11^C]MDL100907 BP_ND_ in the OFC, INS, AMYG, and CAU at uncorrected *p* < 0.05. Motor impulsiveness had a positive correlation with [^11^C]DASB BP_ND_ in the CAU at uncorrected *p* < 0.05. Our results suggest that impulsivity traits, characterized by focusing on the present moment without considering future consequences, may be involved in serotonergic neurotransmission, particularly 5-HT2A receptor-mediated postsynaptic signaling in the CAU, which plays an important role in cognitive processes related to executive function, judgment of alternative outcomes, and inhibitory control.

## 1. Introduction

Impulsivity (or impulsiveness) is a multifaceted personality trait that includes the tendency to act on a whim characterized by little or no consideration of consequences, reflection, or forethought [1]. Thus, impulsive behaviors are typically poorly conceived, prematurely expressed, unduly risky, or inappropriate for the situation, often resulting in undesirable outcomes [2]. It is also a key clinical feature of several psychiatric disorders, including borderline/antisocial personality disorders (BPD/APD), bipolar disorder, substance use disorder, and attention-deficit hyperactivity disorder [3,4,5]. Therefore, understanding the neural mechanisms of impulsivity is important in research and clinical fields regarding risky behaviors and psychiatric disorders.

Impulsivity is strongly related to the intrinsic properties of monoaminergic neurotransmitter systems in the brain, and their networks are believed to be subject to extensive bottom-up modulation by these systems [6]. In particular, the serotonergic system plays an important role in modulating impulsivity [6,7]. Several postmortem studies have shown that suicide victims, who are closely associated with impulsivity, have abnormalities in several serotonergic parameters, such as decreased serotonin (5-hydroxytryptamine, 5-HT) levels [8,9], presynaptic 5-HT transporter (5-HTT) binding [10,11,12], and postsynaptic 5-HT2A receptor binding [13] in the brain, while some studies have shown a decrease in the number of presynaptic 5-HT1A autoreceptors in the dorsal raphe nucleus [9] and in presynaptic 5-HTT and postsynaptic 5-HT2A receptor binding in the prefrontal cortex (PFC) [14,15]. Furthermore, suicide victims have been shown to have a localized decrease in 5-HTT binding in the ventral PFC and fewer 5-HTT messenger ribonucleic acid (mRNA)-expressing neurons in the dorsal raphe nuclei [8,16]. Several clinical studies using positron emission tomography (PET) have reported decreased 5-HTT levels in suicide attempters who are more impulsive [17,18]. Additionally, a PET study on impulsive females with BPD reported increased postsynaptic 5-HT2A receptor availability in the hippocampus [19]. These studies suggest that impulsivity in pathological conditions is associated with presynaptic and/or postsynaptic serotonergic alterations.

As mentioned above, impulsive behaviors are a common clinical feature of BPD, APD, and several other psychiatric disorders, but impulsiveness also has important implications for healthy individuals, as it significantly influences an individual’s social life. Its adverse outcomes can include delinquency, addiction, antisocial behaviors, aggression, pathological gambling, and crimes [20,21,22,23,24]. Impulsivity is also closely associated with other personality constructs, such as sensation seeking [25], failure to plan [26], lack of perseverance [27], venturesomeness [28], poor self-discipline [29], and temperamental traits such as high novelty seeking, low harm avoidance, and low persistence [30,31]. Two studies found that the general population that did not fully meet the diagnostic criteria for BPD exhibited symptoms qualitatively similar to those of patients with the disorder [32,33], suggesting that impulsivity lies along a continuum of personality traits, from normal to pathological. Regarding impulsivity in healthy individuals, a PET study reported a significant positive correlation with 5-HTT binding in the pons in participants composed of only males [34], but several studies have reported no significant correlations with postsynaptic 5-HT2A receptor binding in the frontal cortex or 5-HT1A receptor binding in the cerebral cortex [35,36].

Despite these intriguing results from several studies, direct in vivo evidence of the link between the serotonergic system and impulsivity in humans is still lacking, particularly in healthy individuals. Based on previous findings, it is necessary to explore the specific nature of impulsivity using PET with selective radioligands for presynaptic and postsynaptic serotonergic markers. However, to date, few studies have reported associations between impulsivity levels and presynaptic and postsynaptic serotonergic markers in healthy individuals using double-tracer PET investigation [34]. Therefore, to elucidate the potential roles of presynaptic and postsynaptic 5-HT markers in specific constructs of impulsivity, we investigated whether impulsivity traits (attentional impulsiveness, motor impulsiveness, and non-planning impulsiveness) in healthy individuals are associated with in vivo cerebral 5-HTT and 5-HT2A receptor availability using PET with [^11^C]DASB and [^11^C]MDL100907, controlling for confounding variables. In this study, we chose 5-HTT availability as a presynaptic marker and 5-HT2A receptor availability as a postsynaptic marker based on previous postmortem reports that these serotonergic markers are related to impulsivity [10,11,12,13,14,15].

## 2. Results

The mean scores for the attentional impulsiveness, motor impulsiveness, and non-planning impulsiveness subscales were 14.2 ± 3.1 (range: 9–23), 18.3 ± 3.4 (range: 13–26), and 23.4 ± 4.3 (range: 15–33), respectively (Table 1). The mean total score for BIS-11 was 55.8 ± 8.7 (range: 37–69). The Barratt Impulsiveness Scale-11 (BIS-11) scores were not significantly correlated with age (*r* = −0.17 to −0.09, *p* > 0.05). There were no significant differences between males and females in BIS-11 scores (t = −0.71 to 1.06, *p* > 0.05).

Males had significantly higher in [^11^C]DASB binding potential with respect to the non-displaceable compartment (BP_ND_) in the PFC (t = 3.44, *p* = 0.002), ventromedial PFC (vmPFC) (t = 3.44, *p* = 0.002), orbitofrontal cortex (OFC) (t = 2.24, *p* = 0.035), anterior cingulate cortex (ACC) (t = 3.14, *p* < 0.001), and caudate (CAU) (t = 2.35, *p* = 0.040), and [^11^C]MDL100907 BP_ND_ in the hippocampus (HIP) (t = 2.27, *p* = 0.033) and putamen (PUT) (t = 2.24, *p* = 0.035) than females. Age was significantly negatively correlated with [^11^C]MDL100907 BP_ND_ in the PFC (*r* = −0.54, *p* = 0.005), vmPFC (*r* = −0.52, *p* = 0.007), ACC (*r* = −0.47, *p* = 0.016), and insula (INS) (*r* = −0.47, *p* = 0.016) (Supplementary Tables S1 and S2). Harm avoidance was significantly positively correlated with attentional impulsiveness (*r* = 0.67, *p* < 0.001), and novelty seeking was significantly positively correlated with motor impulsiveness (*r* = 0.62, *p* < 0.001), non-planning impulsiveness (*r* = 0.40, *p* = 0.046), and the total BIS-11 score (*r* = 0.55, *p* = 0.003) (Supplementary Table S3).

**Figure 1:**
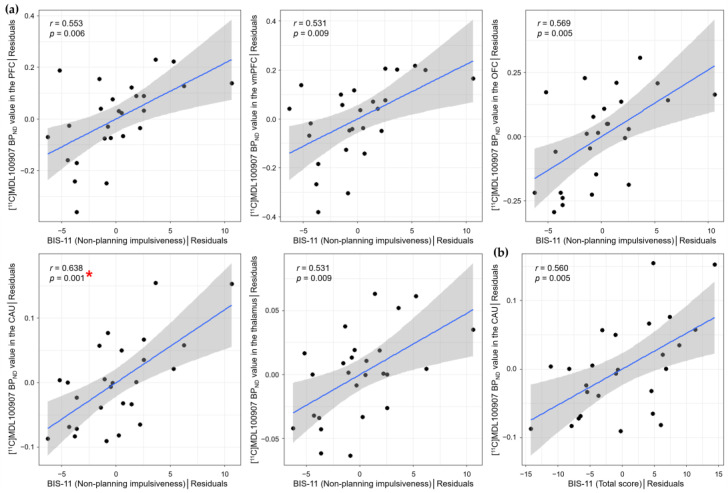
Scatter plots showing the partial correlations between the BIS-11 scores and [^11^C]MDL100907 BP_ND_ in specific brain regions controlling for age, sex, and temperament scores of the TCI. (**a**) Non-planning impulsiveness had positive correlations with [^11^C]MDL100907 BP_ND_ in the PFC (*r* = 0.553, *p* = 0.006), vmPFC (*r* = 0.531, *p* = 0.009), OFC (*r* = 0.569, *p* = 0.005), CAU (*r* = 0.638, *p* = 0.001), and THA (*r* = 0.531, *p* = 0.009). (**b**) The total score of the BIS-11 had a positive correlation with [^11^C]MDL100907 BP_ND_ in the CAU (*r* = 0.560, *p* = 0.005). These results were significant at the thresholds of uncorrected two-tailed *p* < 0.01. The result marked with a red asterisk in *p*-value was significant at the Bonferroni-corrected two-tailed *p* < 0.00455. The black dots represent ordered pairs of the unstandardized residuals estimated from two separate linear regressions of the BIS-11 scores and [^11^C]MDL100907 BP_ND_ in the ROIs in regard to age, sex, and temperament scores that were significantly associated with the BIS-11 scores. The blue solid line and gray area represent the regression line and 95% confidence interval, respectively. BIS-11, Barratt impulsiveness scale-11; BP_ND_, binding potential with respect to non-displaceable compartment; TCI, Temperament, and Character Inventory; PFC, prefrontal cortex; vmPFC, ventromedial prefrontal cortex; OFC, orbitofrontal cortex; CAU, caudate nucleus; THA, thalamus; ROI, region of interest.

The region of interest (ROI)-based partial correlation analysis, with age, sex, and temperaments as covariates, showed a significant positive correlation between non-planning impulsiveness and [^11^C]MDL100907 BP_ND_ in the CAU (*r* = 0.638, *p* = 0.001), which was significant at Bonferroni-corrected *p* < 0.0045 (Table 2, Figure 1a). This analysis also revealed that motor impulsiveness had a positive correlation with [^11^C]DASB BP_ND_ in the CAU (*r* = 0.480, *p* = 0.020) at uncorrected *p* < 0.05 (Table 3). Non-planning impulsiveness was positively correlated with [^11^C]MDL100907 BP_ND_ in the PFC (*r* = 0.553, *p* = 0.006), vmPFC (*r* = 0.531, *p* = 0.009), OFC (*r* = 0.569, *p* = 0.005), INS (*r* = 0.497, *p* = 0.016), amygdala (AMYG) (*r* = 0.500, *p* = 0.015), PUT (*r* = 0.442, *p* = 0.035), ventral striatum (VS) (*r* = 0.417, *p* = 0.048), and thalamus (THA) (*r* = 0.531, *p* = 0.009) (Table 2, Figure 1a), and the total score of BIS-11 had positive correlations with [^11^C]MDL100907 BP_ND_ in the OFC (*r* = 0.494, *p* = 0.017), INS (*r* = 0.415, *p* = 0.049), AMY (*r* = 0.416, *p* = 0.048), and CAU (*r* = 0.560, *p* = 0.005) at uncorrected *p* < 0.05 (Table 2, Figure 1b).

## 3. Discussion

In this study, we found that motor impulsiveness was positively correlated with 5-HTT availability in the CAU and that both non-planning impulsiveness and total BIS scores were positively correlated with 5-HT2A receptor availability in several brain regions of the fronto-striatal circuits, AMYG, and INS. In particular, a significant correlation between non-planning impulsiveness and 5-HT2A receptor availability in the CAU was confirmed by Bonferroni correction for multiple correlations. The results of this study suggest that human impulsivity traits are involved in serotonergic functions in the CAU and its related fronto-striatal circuits and that the non-planning impulsiveness sub-trait is strongly associated with 5-HT2A receptor-mediated postsynaptic signaling. A previous double-tracer PET study conducted in males with high impulsive-aggressive behaviors reported a significant positive correlation between impulsiveness and 5-HTT availability in the pons, but not with 5-HT2A receptor availability [34]. To the best of our knowledge, this is the first double-tracer PET study to report significant associations between impulsivity traits and 5-HT2A receptor availability in healthy males and females.

In the ROI-based correlation analysis controlling for confounding variables, the first main finding was that higher motor impulsiveness was associated with higher 5-HTT availability in the CAU and that higher non-planning impulsiveness and total BIS-11 scores were associated with higher 5-HT2A receptor availability in the CAU. The dorsal striatum, which consists of the CAU, has been implicated in response inhibition [37,38,39] and motor control [40]. Furthermore, a resting-state functional MRI study using graph theory analyses revealed a significant relationship between impulsivity levels and brain network regions consisting of the CAU and its related cortical areas in healthy individuals [41]. Several structural magnetic resonance imaging (MRI) studies have also reported that volumetric changes in the CAU are associated with behavioral aspects characterized by behavioral control deficits and impulsivity traits, including attentional and motor impulsiveness [42,43,44]. Therefore, based on these previous studies, our results suggest that serotonergic signaling in the CAU may be linked to impulsivity traits, i.e., inhibition of motor reactions and forward-thinking. These impulsivity traits could be mainly caused by altered 5-HT levels with secondary changes in 5-HTT and 5-HT2A receptor density, or by genetic factors that affect presynaptic and postsynaptic serotonergic markers.

Notably, our study found that the positive correlation between non-planning impulsiveness levels and 5-HT2A receptor availability in the CAU survived Bonferroni correction for multiple correlations (*p* < 0.00455). Although there have been no previous studies on the relationship between impulsiveness sub-traits and in vivo serotonergic markers, our results suggest that a non-planning impulsivity sub-trait in healthy individuals may be closely associated with postsynaptic rather than presynaptic serotonergic signaling.

The second main finding of our study was that non-planning impulsiveness was positively correlated with 5-HT2A receptor availability in the PFC, vmPFC, OFC, PUT, VS, and THA, and the total BIS-11 score was positively correlated with 5-HT2A receptor availability in the OFC at uncorrected *p* < 0.05. These brain regions are part of the fronto-striatal circuit, and the dysregulation of this circuit has been suggested as a neural substrate of impulsivity in preclinical and clinical studies [6,7,45]. A preclinical study using a five-choice serial reaction time task (5-CSRTT) showed that premature responses to 5-CSRTT were positively associated with tonic extracellular 5-HT levels in the PFC [46], suggesting that altered stimulation of 5-HT receptors in the PFC may disrupt the inhibitory response control [47]. Several neuroimaging studies have shown that structural changes in the prefrontal regions and striatum are associated with impulsive behaviors in healthy individuals [48,49]. Our findings support the notion that impulsivity traits are related to fronto-striatal functional activity in the serotonergic system [6,7,50,51].

We also found that both non-planning impulsiveness and total BIS-11 scores were positively correlated with 5-HT2A receptor availability in the INS and AMYG at uncorrected *p* < 0.05. These results suggest a possibility that postsynaptic serotonergic signaling in these regions is associated with affective aspects related to impulsivity. Our results are in line with previous reports that impulsive behaviors are involved in alterations in affective processing and that functional connectivity consisting of the INS and AMYG is associated with impulsivity traits [52].

In our study, motor impulsiveness showed a statistical tendency towards a positive correlation, and only the correlation between non-planning impulsiveness and [^11^C]MDL100907 BP_ND_ in the CAU showed a significant positive correlation which survived the Bonferroni correction for multiple correlations. Therefore, we suggest that only a non-planning impulsiveness trait may be closely associated with 5-HT2A receptor-mediated postsynaptic signaling in the CAU. It is unclear whether the positive correlation means lower 5-HT signaling cascades underlying high non-planning impulsiveness. Since [^11^C]MDL100907, an antagonist tracer, binds to both high- and low-affinity 5-HT2A receptors located on the postsynaptic neurons, subtle changes in endogenous 5-HT levels are not assumed to be reflected by [^11^C]MDL100907 BP_ND_ [53]. However, we cannot rule out the possibility that 5-HT2A availability reflects the result of compensatory regulation due to the 5-HT2A receptor occupancy status caused by endogenous 5-HT [54]. Chronically low levels of 5-HT may induce a compensatory up-regulation of receptors (increased Bmax). Future studies using agonist tracers for 5-HT2A receptors (e.g., [^11^C]CIMBI-5) are needed to clarify this issue as agonist tracers bind preferentially to receptors in the high-affinity state providing enhanced sensitivity to competition with endogenous 5-HT [55]. Moreover, corresponding specific intracellular responses to 5-HT2A receptor-mediated activation should be explored at a more fundamental level by measuring gene expression and its protein products in the future with the development of adequate in vivo probes. In addition, further double-tracer PET studies are required to investigate interactions with other neurotransmitter systems and to gain more insights into the relationships between impulsivity traits and various neurotransmitters (e.g., dopamine and 5-HT).

In this study, 3-Tesla MRI and PET were used to obtain imaging data. Compared to 1.5-Tesla MRI or CT, 3-Tesla MRI has better contrast among soft tissues and provides functional-imaging capabilities. Therefore, 3-Tesla MRI and PET have advantages in combining functional PET and structural MRI information. The 3-Tesla MRI and PET systems are promising tools in clinical applications, and many research studies, including our own, are underway in the fields of neurology and psychiatry.

The interpretation of the results of our study should be considered in light of several limitations. Due to a relatively small sample size, our PET study is considered a preliminary study although the number of participants who completed double tracer PET imaging is quite similar to that of previous PET studies [34,56]. It is possible that our study was underpowered to detect significant correlations. Further larger studies are warranted to confirm our findings. The limited range of BIS-11 scores in healthy individuals can limit the detection of significant correlations with presynaptic and postsynaptic serotonergic parameters. We chose in vivo 5-HT2A receptor availability as a potential postsynaptic marker implicated in impulsivity traits. However, other 5-HT receptors such as 5-HT1A and 5-HT4 may also play a role in impulsiveness for healthy individuals [57,58,59], suggesting that complex dynamics of 5-HT subsystems may be associated with impulsivity traits. Further PET molecular imaging studies on specific 5-HT subsystems are needed to unravel the 5-HT system associated with various aspects of impulsivity traits. In this study, [^11^C]DASB and [^11^C]MDL100907 BP_ND_ were estimated using the simplified reference tissue model 2 (SRTM2) [60] and the basis function implementation of the SRTM [57,61]. However, a two-tissue compartment model (2TCM) with an arterial input function (AIF) is the optimal kinetic model. In the kinetic model for 2TCM, AIF is obtained using invasive methods, such as radial artery cannulation, and the resulting discomfort and inaccuracies in determining AIF could be a source of bias in endpoint estimation [62]. Therefore, in our study, we computed [^11^C]DASB and [^11^C]MDL100907 BP_ND_ values using the SRTM2 and the basis function implementation of SRTM, respectively, with the cerebellum as a reference region without specific binding, as previously suggested for these tracers [57,63,64,65,66,67,68]. In the present study, imaging data were obtained using 3-Tesla MRI and PET. To elucidate the 5-HT system involved in different aspects of impulsivity traits based on imaging data with high spatial resolution, further studies using ultra-high-resolution MRI and PET are needed.

## 4. Materials and Methods

### 4.1. Participants

The study protocol was approved by the Institutional Review Board of the Gachon University Gil Medical Center (GBIRB2020-275) and all study procedures were conducted in accordance with international ethical standards and the Declaration of Helsinki. All subjects provided written informed consent after a full explanation of the study‘s purpose and procedures prior to participating in the study.

Twenty-six healthy subjects (10 males and 16 females) were recruited through advertisements on local posters. The inclusion criteria were as follows: (i) aged 19 to 60 years; (ii) no past or current psychiatric disorders from the Diagnostic and Statistical Manual of Mental Disorders 4th edition (DSM-IV) [69], established using the Structured Clinical Interview for DSM-IV [70]; (iii) no past or current substance dependence/use; (iv) no history of neurological or medical disorders; (v) no past or current use of substances/medications known to affect the central nervous system; and (vi) not pregnant at the date of the PET scan. Their mean age was 31.4 ± 8.4 years (range = 21–50 years, median = 29 years) and the mean duration of education was 15.7 ± 0.7 years (Table 1). A board-certified radiologist confirmed that none of the subjects had structural abnormalities on MRI. Demographic information on the subjects is presented in Table 1.

### 4.2. Assessment of Impulsivity

The BIS-11 is a self-report questionnaire that measures the personality/behavioral construct of impulsiveness and consists of three subscales: attentional impulsiveness (inattention and cognitive instability), motor impulsiveness (spontaneous actions), and non-planning impulsiveness (lack of forethought) [26]. In this study, the participants’ levels of these traits were assessed using the standardized Korean version of the BIS-11-Revised [71], which is a 30-item self-administered questionnaire, with each item rated on a 4-point scale (1, rarely/never to 4, almost always). Higher BIS-11 scores indicate higher levels of impulsiveness.

The Temperament and Character Inventory (TCI) [31] was used to control for the potentially confounding effects of general temperaments (i.e., harm avoidance, novelty seeking, reward dependence, and persistence) that are conceptually or empirically related to monoaminergic neurotransmission. In this study, participants’ levels of these temperaments were assessed using the standardized Korean version of the TCI-Revised [72], which is a 140-item questionnaire, with each item rated on a 5-point scale (0, not at all to 4, very true).

### 4.3. Scan Protocol

All subjects were scanned for both [^11^C]DASB and [^11^C]MDL100907 using a Biograph 6 PET scanner (Siemens Medical Imaging Systems, Knoxville, TN, USA). A computed tomography (CT)-based transmission scan was performed immediately before tracer injection for attenuation correction. After each bolus injection of 751.5 ± 68.3 MBq [^11^C]DASB with an average specific activity of 64.3 ± 28.7 GBq/μmol and 693.6 ± 54.5 MBq [^11^C]MDL100907 with an average specific activity of 61.8 ± 34.1 GBq/μmol (Table 1), emission data were acquired in dynamic mode for 90 min. The two PET scans obtained for each subject were acquired with an average gap between scans of 64 ± 46 days. The emission data were reconstructed using the two-dimensional ordered-subset expectation maximization (OSEM-2D) algorithm into 22 frames of the following durations: 4 × 30 s, 2 × 60 s, 2 × 90 s, 3 × 150 s, 3 × 210 s, 4 × 300 s, 3 × 600 s, and 1 × 900 s. The reconstructed PET images had a voxel size of 1.33 × 1.33 × 1.50 mm^3^ and a matrix size of 256 × 256 × 109. These PET frames were corrected for random and scatter coincidences, attenuation, detector dead time, decay, and detector normalization.

Structural MRI data were acquired using a 3-Tesla MRI scanner (Magnetom Vida; Siemens Healthcare, Erlangen, Germany) with a 20-channel receiver head/neck coil. In this study, a three-dimensional T1-weighted magnetization-prepared rapid gradient echo (3D T1MPRAGE) sequence was used and had the following scan parameters: repetition time = 1800 ms, echo time = 2.61 ms, inversion time = 900 ms, flip angle = 10°, voxel size = 0.5 × 0.5 × 1.0 mm^3^, matrix size = 512 × 416, and number of slices = 176. During the PET and MRI scans, the heads of the participants were held as comfortably as possible using sponges to minimize head movements.

### 4.4. Image Analysis

**Figure 2:**
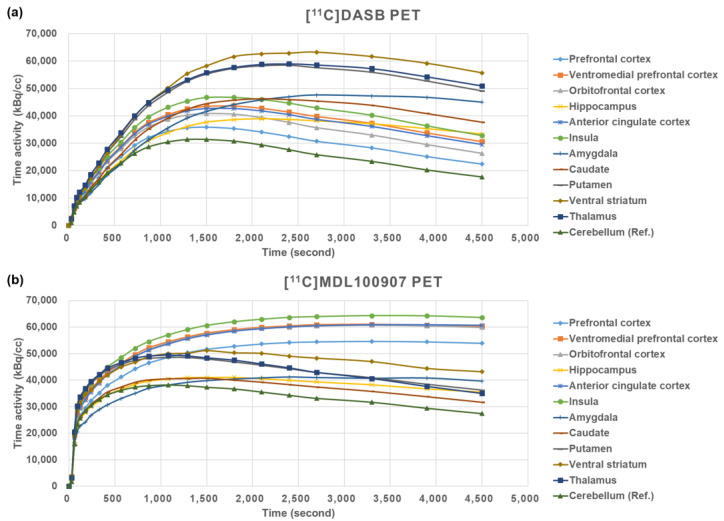
Average TACs of [^11^C]DASB and [^11^C]MDL100907 in healthy individuals. These TACs were extracted from each ROI in each reconstructed 22 PET frame of the [^11^C]DASB (**a**) and [^11^C]MDL100907 (**b**). The [^11^C]DASB BP_ND_ and [^11^C]MDL100907 BP_ND_ of each ROI were obtained from the TACs with the cerebellum as the reference region. TAC, time-activity curve; ROI, region of interest; BP_ND_, binding potential with respect to non-displaceable compartment; PET, positron emission tomography.

**Figure 3:**
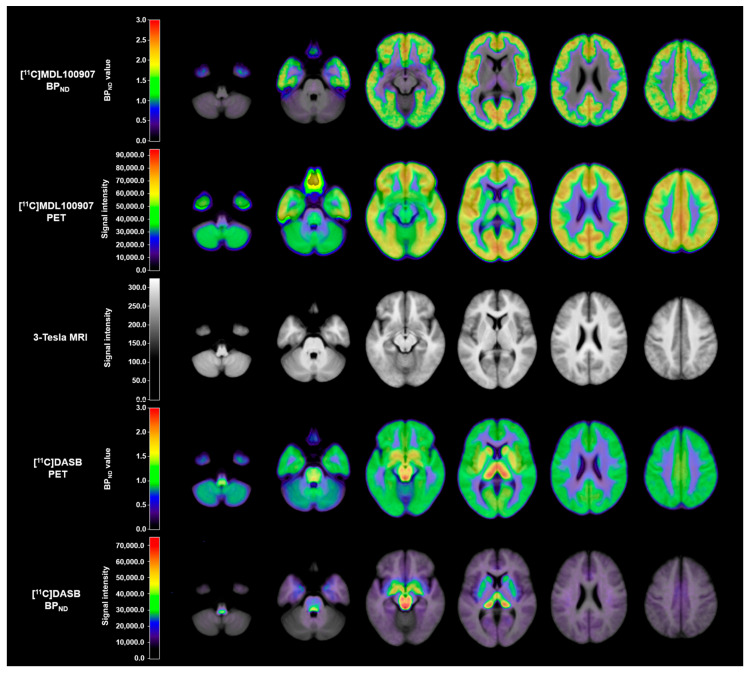
Representative average images of [^11^C]MDL100907 BP_ND_, [^11^C]MDL100907 PET, [^11^C]DASB BP_ND_, [^11^C]DASB PET, and corresponding 3-Tesla MRI in healthy individuals. BP_ND_, binding potential with respect to non-displaceable compartment; PET, positron emission tomography; MRI, magnetic resonance imaging.

For each [^11^C]DASB and [^11^C]MDL100907 PET dataset, image preprocessing was performed using Statistical Parametric Mapping 12 (SPM12; The Wellcome Center for Human Neuroimaging, London, UK; www.fil.ion.ucl.ac.uk (accessed on 1 October 2014)). Realignment was conducted for motion correction within all reconstructed PET frames. Each subject’s structural MRI image was coregistered with their own mean PET image derived from the realignment step. The coregistered structural MRI images were spatially normalized using the Montreal Neurological Institute (MNI) template with a nonlinear deformation field, and the estimated transform was applied to the corresponding PET frames. Based on parameter estimation by kinetic modeling implemented in PMOD software v4.2 (PMOD Technologies Ltd., Zürich, Switzerland), the BP_ND_ images of each [^11^C]DASB and [^11^C]MDL100907 were derived using the SRTM2 [60] and the basis function implementation of the SRTM [57,73,74] with cerebellar gray matter as a reference region, as suggested in previous studies on these tracers [57,63,64,65,66,67,68]. In each [^11^C]DASB and [^11^C]MDL100907 BP_ND_ estimation step, regional time-activity curves (TACs) were extracted from spatially normalized PET frames by averaging all voxels within 11 a priori ROIs related to the serotonergic system and impulsive behaviors, based on previous studies [36,75,76] (Figure 2). These ROIs included the PFC, vmPFC, OFC, ACC, HIP, INS, AMYG, CAU, PUT, VS, and THA. The PFC includes the dorsolateral prefrontal, medial prefrontal, ventrolateral prefrontal, and orbitofrontal subregions [77], and its anatomical location was determined based on the Brodmann areas (BA) in the Talairach atlas [78]. Of these, seven ROIs were predefined using the automated anatomical labeling atlas 3 (AAL3) [79], excluding the PFC and its subregions predefined by the BA, and the thalamus predefined by the AAL [80]. Representative examples of [^11^C]MDL100907 BP_ND_, [^11^C]MDL100907 PET, [^11^C]DASB BP_ND_, [^11^C]DASB PET, and the corresponding 3-Tesla MRI images are shown in Figure 3.

### 4.5. Statistical Analysis

Several studies have shown that age and sex affect 5-HTT [81,82] and 5-HT2A receptor availability [83,84] and that sex affects impulsivity [85,86]. To investigate sex differences in [^11^C]DASB BP_ND_ and [^11^C]MDL100907 BP_ND_ in each ROI, the Shapiro–Wilk test and Levene’s test were first performed to assess normality and the equality of variances, and *p* values less than 0.05 were considered a significant deviation from normality. Independent samples *t*-tests were used to compare the mean values of [^11^C]DASB BP_ND_ and [^11^C]MDL100907 BP_ND_ between males and females in each ROI. An uncorrected two-tailed *p* < 0.05 level was considered statistically significant. In our sample, significant sex differences were found in [^11^C]DASB BP_ND_ in some brain regions, and significant negative correlations were found between age and [^11^C]MDL100907 BP_ND_ in several regions of the brain (Supplementary Tables S1–S3). To evaluate the potentially confounding effects of temperaments, we used Pearson’s correlation analysis to examine the relationship between impulsivity and temperament traits. These analyses showed that impulsivity in our sample was significantly correlated with temperaments (attentional impulsiveness and harm avoidance, motor impulsiveness, and novelty seeking, non-planning impulsiveness and novelty seeking, and total BIS and novelty seeking scores (Supplementary Table S4). Therefore, ROI-based partial correlation analyses were performed to explore the relationships between the BIS-11 scores and [^11^C]DASB and [^11^C]MDL100907 BP_ND_ in different brain regions, controlling for the effects of age, sex, and temperament scores that were significantly associated with the BIS scores. The level of statistical significance was defined as the Bonferroni-corrected two-tailed *p* < 0.00455 (0.05/11) for multiple correlations (11 brain regions), and the level of uncorrected two-tailed *p* < 0.05 was considered a statistical tendency. All statistical analyses were performed using the Statistical Package for the Social Sciences (SPSS) v28.0 (IBM Corp., Armonk, NY, USA).

## 5. Conclusions

Our results suggest that impulsivity traits, characterized by focusing on the present moment without considering future consequences, are involved in serotonergic neurotransmission, particularly 5-HT2A receptor-mediated signaling in the CAU, which plays an important role in cognitive processes related to executive function, judgment of alternative outcomes, and inhibitory control. Due to a relatively small sample size, our PET study is considered a preliminary study. Further larger studies are warranted to confirm our findings.

## Figures and Tables

**Table 1:** Demographic and PET scan information.

Variables		Mean ± SD/Number (%)
Demographic information		
Age (years)		31.4 ± 8.4
Sex		
Male		10 (38.5%)
Female		16 (61.5%)
Education (years)		15.7 ± 0.7
Barratt impulsiveness scale-11		
Attentional impulsiveness		14.2 ± 3.1
Motor impulsiveness		18.3 ± 3.4
Non-planning impulsiveness		23.4 ± 4.3
Total score		55.8 ± 8.7
TCI temperament scale		
Novelty seeking		30.5 ± 9.9
Harm avoidance		33.5 ± 13.4
Reward dependence		45.0 ± 8.8
Persistence		40.7 ± 8.8
PET scan information		
[^11^C]DASB	Injected dose (MBq)	751.5 ± 68.3
	Specific activity (GBq/μmol)	64.3 ± 28.7
[^11^C]MDL100907	Injected dose (MBq)	693.6 ± 54.5
	Specific activity (GBq/μmol)	61.8 ± 34.1

PET, positron emission tomography; SD, standard deviation; TCI, Temperament and Character Inventory.

**Table 2:** Average [^11^C]MDL100907 BP_ND_ values and their correlation coefficients with BIS-11 scores in ROIs.

Regions of Interest	Mean (SD)	Correlation Coefficients ^†^ (*p*-Value)			
		Attentional Impulsiveness	Motor Impulsiveness	Non-Planning Impulsiveness	Total Score
Prefrontal cortex	1.27 (0.18)	0.286 (0.186)	0.145 (0.509)	0.553 (0.006) **	0.411 (0.051)
Ventromedial prefrontal cortex	1.40 (0.19)	0.222 (0.309)	0.176 (0.421)	0.531 (0.009) **	0.394 (0.063)
Orbitofrontal cortex	1.33 (0.19)	0.307 (0.154)	0.179 (0.415)	0.569 (0.005) **	0.494 (0.017) *
Hippocampus	0.42 (0.07)	0.329 (0.125)	0.299 (0.166)	0.362 (0.089)	0.392 (0.064)
Anterior cingulate cortex	1.30 (0.18)	0.145 (0.508)	0.077 (0.726)	0.383 (0.071)	0.258 (0.234)
Insula	1.42 (0.18)	0.322 (0.133)	0.122 (0.580)	0.497 (0.016) *	0.415 (0.049) *
Amygdala	0.64 (0.10)	0.247 (0.255)	0.229 (0.293)	0.500 (0.015) *	0.416 (0.048) *
Caudate	0.28 (0.07)	0.310 (0.150)	0.139 (0.528)	**0.638 (0.001)** **^¥^**	0.560 (0.005) **
Putamen	0.29 (0.06)	0.151 (0.491)	0.257 (0.237)	0.442 (0.035) *	0.339 (0.113)
Ventral striatum	0.57 (0.11)	0.068 (0.759)	0.203 (0.353)	0.417 (0.048) *	0.392 (0.064)
Thalamus	0.23 (0.04)	0.042 (0.847)	0.073 (0.740)	0.531 (0.009) **	0.348 (0.1)

^†^ Correlation coefficients were estimated using partial correlation analysis with age, sex, and temperament scores of the TCI as covariates. Asterisks and bold represent statistical significance at *p* < 0.05 * and *p* < 0.01 **. ^¥^ This result is significant at Bonferroni-corrected *p* < 0.00455. BP_ND_, binding potential with respect to non-displaceable compartment; BIS-11, Barratt Impulsiveness Scale-11; ROI, region of interest; SD, standard deviation; TCI, Temperament, and Character Inventory.

**Table 3:** Average [^11^C]DASB BP_ND_ values and their correlation coefficients with BIS-11 scores in ROIs.

Regions of Interest	Mean (SD)	Correlation Coefficients ^†^ (*p*-Value)			
		Attentional Impulsiveness	Motor Impulsiveness	Non-Planning Impulsiveness	Total Score
Prefrontal cortex	0.38 (0.06)	−0.147 (0.504)	0.366 (0.086)	−0.145 (0.508)	−0.013 (0.954)
Ventromedial prefrontal cortex	0.57 (0.11)	−0.136 (0.535)	0.390 (0.065)	−0.178 (0.416)	−0.038 (0.863)
Orbitofrontal cortex	0.45 (0.07)	−0.075 (0.734)	0.364 (0.088)	−0.103 (0.640)	0.046 (0.835)
Hippocampus	0.83 (0.16)	−0.157 (0.474)	0.272 (0.209)	−0.281 (0.194)	−0.202 (0.354)
Anterior cingulate cortex	0.46 (0.10)	−0.075 (0.733)	0.398 (0.060)	−0.181 (0.407)	−0.019 (0.930)
Insula	0.66 (0.13)	−0.080 (0.716)	0.335 (0.118)	−0.226 (0.301)	−0.046 (0.834)
Amygdala	1.84 (0.28)	−0.087 (0.693)	−0.037 (0.867)	−0.095 (0.665)	−0.108 (0.623)
Caudate	1.04 (0.27)	−0.019 (0.930)	0.480 (0.020) *	0.019 (0.931)	0.101 (0.647)
Putamen	1.50 (0.32)	−0.142 (0.517)	0.378 (0.075)	−0.073 (0.739)	0.026 (0.906)
Ventral striatum	1.81 (0.39)	−0.197 (0.369)	0.168 (0.443)	−0.026 (0.907)	−0.069 (0.753)
Thalamus	1.61 (0.24)	−0.001 (0.998)	0.402 (0.057)	0.244 (0.262)	0.283 (0.190)

^†^ Correlation coefficients were obtained using partial correlation analysis with age, sex, and temperament scores of the TCI as covariates. Asterisks and bold indicate statistical significance at *p* < 0.05. BP_ND_, binding potential with respect to non-displaceable compartment; BIS-11, Barratt Impulsiveness Scale-11; ROI, region of interest; SD, standard deviation; TCI, Temperament, and Character Inventory.

## Data Availability

The data presented in this study are available upon reasonable request from the corresponding authors.

## Supplementary Materials

The following supporting information can be downloaded at https://www.mdpi.com/article/10.3390/ijms26010252/s1.
