# Supplementary material for: The Relationship Between Impulsivity Traits and In Vivo Cerebral Serotonin Transporter and Serotonin 2A Receptor Binding in Healthy Individuals: A Double-Tracer PET Study with C-11 DASB and C-11 MDL100907
Source: Int J Mol Sci. 2024 Dec 30;26(1):252. doi: 10.3390/ijms26010252 (PMC11720673; doi:10.3390/ijms26010252)
Supplement: Supplementary file 1 [file ijms-26-00252-s001.zip › ijms-3381010-supplementary.pdf]

Supplementary Materials

Supplementary Table S1. Comparison between males and females for regional [^{11}C]DASB BP_{ND}

Brain region	[^{11}C]DASB BP_{ND}					
	Males (n = 10)	Females (n = 16)	Levene's test		Independent samples *t*-test	
			F value	*p*-value	*t* value	*p*-value
Prefrontal cortex	0.43 ± 0.07	0.35 ± 0.04	3.66	0.068	3.44	0.002**
Ventromedial prefrontal cortex	0.64 ± 0.13	0.52 ± 0.06	4.07	0.055	3.44	0.002**
Orbitofrontal cortex	0.49 ± 0.09	0.43 ± 0.05	4.19	0.052	2.24	0.035*
Hippocampus	0.88 ± 0.21	0.79 ± 0.10	6.91	0.015	1.28	0.226
Anterior cingulate cortex	0.54 ± 0.12	0.41 ± 0.05	6.85	0.015	3.14	<0.010**
Insula	0.72 ± 0.18	0.62 ± 0.07	7.93	0.010	1.77	0.106
Amygdala	1.89 ± 0.30	1.81 ± 0.27	0.19	0.67	0.69	0.496
Caudate nucleus	1.20 ± 0.35	0.93 ± 0.13	8.96	0.006	2.35	0.040*
Putamen	1.66 ± 0.41	1.40 ± 0.20	5.67	0.026	1.84	0.091
Ventral striatum	2.00 ± 0.47	1.69 ± 0.27	6.24	0.020	1.83	0.091
Thalamus	1.68 ± 0.26	1.67 ± 0.22	1.00	0.327	1.22	0.234

[^{11}C]DASB BP_{ND} values are indicated as mean ± standard deviation. In these analysis, the statistical significance is indicated as $p < 0.05$* and $p < 0.01$**. BP_{ND}, binding potential with respect to non-displaceable compartment.

Supplementary Table S2. Comparison between males and females for regional [^{11}C]MDL100907 BP_{ND}

Brain region	[^{11}C]MDL100907 BP_{ND}					
	Males (n = 10)	Females (n = 16)	Levene's test		Independent samples *t*-test	
			F value	*p*-value	*t* value	*p*-value
Prefrontal cortex	1.27 ± 0.18	1.27 ± 0.19	0.09	0.770	-0.02	0.986
Ventromedial prefrontal cortex	1.38 ± 0.17	1.41 ± 0.20	0.86	0.363	-0.40	0.692
Orbitofrontal cortex	1.30 ± 0.19	1.35 ± 0.20	0.24	0.631	-0.61	0.550
Hippocampus	0.46 ± 0.08	0.40 ± 0.06	0.45	0.508	2.27	0.033*
Anterior cingulate cortex	1.28 ± 0.15	1.31 ± 0.19	0.85	0.365	-0.46	0.652
Insula	1.42 ± 0.16	1.43 ± 0.19	0.11	0.741	-0.16	0.878
Amygdala	0.65 ± 0.11	0.64 ± 0.09	0.19	0.664	0.31	0.762
Caudate nucleus	0.30 ± 0.07	0.27 ± 0.07	0.04	0.845	0.88	0.389
Putamen	0.32 ± 0.08	0.27 ± 0.05	2.78	0.108	2.24	0.035*
Ventral striatum	0.69 ± 0.11	0.55 ± 0.11	0.02	0.894	0.98	0.337
Thalamus	0.24 ± 0.04	0.23 ± 0.03	1.81	0.191	0.59	0.559

[^{11}C]MDL100907 BP_{ND} values are indicated as mean ± standard deviation. In these analysis, the statistical significance is indicated as $p < 0.05$*. BP_{ND}, binding potential with respect to non-displaceable compartment.

Supplementary Table S3. The relationship between age and regional [^{11}C]DASB and [^{11}C]MDL100907 BP_{ND}

Brain region	[^{11}C]DASB BP_{ND} (Mean ± SD)	Correlation coefficient (*p*-value)	[^{11}C]MDL100907 BP_{ND} (Mean ± SD)	Correlation coefficient (*p*-value)
Prefrontal cortex	0.38 ± 0.06	0.147 (0.473)	1.27 ± 0.18	-0.539 (0.005**)
Ventromedial prefrontal cortex	0.57 ± 0.11	0.165 (0.422)	1.40 ± 0.19	-0.517 (0.007**)
Orbitofrontal cortex	0.45 ± 0.07	0.052 (0.799)	1.33 ± 0.19	-0.387 (0.051)
Hippocampus	0.83 ± 0.16	0.140 (0.496)	0.42 ± 0.07	-0.048 (0.815)
Anterior cingulate cortex	0.46 ± 0.10	0.180 (0.378)	1.30 ± 0.18	-0.468 (0.016*)
Insula	0.66 ± 0.13	0.096 (0.640)	1.42 ± 0.18	-0.469 (0.016*)
Amygdala	1.84 ± 0.28	0.188 (0.358)	0.64 ± 0.10	0.184 (0.369)
Caudate nucleus	1.04 ± 0.27	0.276 (0.173)	0.28 ± 0.07	0.064 (0.756)
Putamen	1.50 ± 0.32	0.294 (0.145)	0.29 ± 0.06	0.272 (0.179)
Ventral striatum	1.81 ± 0.39	0.299 (0.138)	0.57 ± 0.11	-0.139 (0.499)
Thalamus	1.61 ± 0.24	-0.009 (0.967)	0.23 ± 0.04	0.331 (0.098)

Correlation coefficients and *p*-values were computed using Pearson's correlation analysis, and the statistical significance is indicated as $p < 0.05$* and $p < 0.01$**. BP_{ND}, binding potential with respect to non-displaceable compartment; SD, standard deviation.

Supplementary Table S4. Correlations between impulsivity and temperament traits

TCI / BIS-11	Correlation coefficient (*p*-value)			
	Noverlty seeking	Harm avoidance	Reward dependence	Persistence
Attentional impulsiveness	0.326 (0.104)	0.669 (<0.001**)	-0.369 (0.064)	-0.266 (0.188)
Motor impulsiveness	0.623 (<0.001**)	0.042 (0.837)	-0.037 (0.858)	-0.266 (0.189)
Non-planning impulsiveness	0.395 (0.046*)	0.138 (0.502)	-0.244 (0.230)	-0.338 (0.091)
Total score	0.553 (0.003**)	0.321 (0.110)	-0.264 (0.192)	-0.364 (0.068)

Correlation coefficients and *p*-values were estimated using Pearson's correlation analysis, and the statistical significance is presented as $p < 0.05$* and $p < 0.01$**. TCI, Temperament and Character Inventory; BIS-11, Barratt Impulsiveness Scale-11.
